# Fostering sustainable food consumption: a theoretical framework for upcycled foods

**DOI:** 10.3389/fpsyg.2025.1682850

**Published:** 2025-10-28

**Authors:** Xi-Yu Zhang, Ching-Tzu Chao, Han-Shen Chen

**Affiliations:** ^1^Department of Accounting, School of Business, Jiaxing University, Jiaxing, China; ^2^Department of Health Industry Technology Management, Chung Shan Medical University, Taichung, Taiwan; ^3^Department of Medical Management, Chung Shan Medical University Hospital, Taichung, Taiwan

**Keywords:** sustainable food consumption, social media advertising, consumer values, consumer decision-making, environmental benefits

## Abstract

**Purpose:**

This study investigates the psychological, social, and environmental determinants of consumers' purchasing intentions for upcycled foods by integrating Value-Belief-Norm Theory (VBNT), Social Cognitive Theory (SCT), and Self-Determination Theory (SDT) into a multi-theoretical framework.

**Design/methodology/approach:**

A survey of 402 online respondents was conducted using convenience sampling, and partial least squares structural equation modeling (PLS-SEM) was used to analyze the results.

**Findings:**

This study found that biospheric and altruistic values drive purchase intentions, enhancing consumers' sense of responsibility and the environmental and social benefits of upcycled food. Consequence awareness was identified as a critical mediator bridging consumers' values and attitudes toward upcycled food consumption. Social media advertising has been found to be a powerful external stimulus that strengthens consumers' self-efficacy, value identification, autonomy, and control motivation.

**Originality:**

This study contributes to the literature on sustainable consumption by demonstrating a multitheoretical approach to unravel consumer decision-making processes.

**Research limitations/implications:**

This study was based solely on a Taiwanese sample, where cultural characteristics (e.g., collectivism and the dominance of the Line app) may limit the generalizability of the findings, highlighting the need for cross-cultural research in this area. This study underscores the importance of strategies that align with values, enhance self-efficacy, and leverage social norms.

**Practical implications:**

The results of this study have practical implications for policymakers, marketers, and industry practitioners, who should develop strategies that emphasize the environmental and social benefits of upcycled foods while addressing consumers' motivational needs for autonomy and efficacy.

**Social implications:**

This study provides insights into fostering sustainable food consumption at the intersection of individual values, social norms, and marketing-driven stimuli.

## Introduction

The Intergovernmental Panel on Climate Change (IPCC) warns that the Earth's temperature is near 1.5 °C above pre-industrial levels (Intergovernmental Panel on Climate Change (IPCC)., 2022). The food system is a major source of greenhouse gases, contributing to over one-third of global emissions [Food Agriculture Organization (FAO)., 2021]. According to the United Nations Environment Programme (UNEP), approximately 1 billion tons of food is wasted annually, with 17% of this waste occurring at the retail and consumer stages (United Nations Environment Programme (UNEP)., 2021). This wasted food could feed 1.26 billion people annually, highlighting the need to improve food security and sustainability in this sector.

To address these challenges, the 28th United Nations Climate Change Conference (COP28) introduced an Agricultural Day to demonstrate how sustainable farming can help combat climate change. This declaration supports the United Nations' Sustainable Development Goals 13, “Climate Action” and 2, “Zero Hunger,” emphasizing the importance of sustainable food production in changing consumption habits and combating climate change.

As the world deals with climate change, food security, and sustainable development, the use of circular economic strategies and promotion of upcycled foods are becoming increasingly crucial. Studies indicate that circular economy strategies can make production and consumption more sustainable (Yang et al., 2023). These strategies involve using waste from the food industry in the food supply chain. This reduces environmental damage and waste, leading to upcycled food products (Ouro-Salim and Guarnieri, 2022).

The Upcycled Food Association (UFA) states that upcycled food is made from ingredients that would otherwise be wasted. These ingredients are sourced and produced through verified supply chains to improve the environment (Upcycled Foods Definition Task Force., 2020). This method not only uses resources better but also supports new business models in the food industry, making it more resilient and sustainable than traditional methods.

Taiwan has made significant strides in its upcycled food sector. FamilyMart and SUNMAI Golden Three Malt collaborated to utilize surplus items, such as bread crusts and malt dregs, resulting in the creation of innovative products, such as eco-friendly beer, honey lemon bread, and malted milk toast. As investment in this sector increases, upcycled food is expected to become more prevalent, thereby contributing to environmental conservation and promoting sustainable food practices.

Despite increasing interest in sustainable food practices, consumer adoption of upcycled food remains constrained by concerns related to safety, quality, and taste, as well as a limited understanding of the upcycling concept (Moshtaghian et al., 2021; Pinela et al., 2024). Existing research has predominantly employed singular theoretical frameworks to investigate consumer perceptions of sustainable foods (Chang et al., 2024).

However, no single theory can fully encapsulate the multifaceted factors that influence consumer choices. For example, the Value-Belief-Norm Theory (VBNT) addresses personal values and moral norms, but inadequately considers motivational aspects. In contrast, Self-Determination Theory (SDT) emphasizes motivation, elucidating why individuals may intend to act but fail to do so. Social Cognitive Theory (SCT) incorporates environmental factors and offers a more comprehensive analysis.

Currently, there is still a lack of in-depth understanding of the underlying drivers that influence consumers' intentions to adopt upcycled foods. This knowledge gap is critical as consumers' environmental awareness continues to increase, yet their acceptance of upcycled foods remains relatively low, creating a paradox that requires further investigation. Previous studies have mostly employed a single theoretical perspective, resulting in fragmented insights that fail to capture the complexity of consumer decision-making in this emerging field of study. Therefore, the main motivation of this study is to address this gap by constructing an integrated theoretical framework that incorporates values, motivations, and social influences, thereby providing a more comprehensive understanding of why consumers choose upcycled food products.

This study aims to achieve three primary objectives: (1) to integrate VBNT, SCT, and SDT to identify the factors influencing consumer intentions and elucidate the psychological and behavioral foundations of purchasing decisions. This integration enhances existing theoretical frameworks and assists businesses in understanding consumer motivation, thereby facilitating the development of more effective marketing strategies. (2) Employing Partial Least Squares Structural Equation Modeling (PLS-SEM) to explore the interconnections among theories and identify the primary determinants of consumer intentions. This methodological approach aids businesses in identifying critical factors and refining their product design and marketing strategies. (3) To investigate consumer acceptance of upcycled food by analyzing the psychological motivations and social norms that influence behavior. This study provides businesses and policymakers with insight into the promotion of upcycled food.

The findings of this study indicate that biospheric and altruistic values play a crucial role in driving consumers' purchase intentions toward upcycled foods. Consequence awareness serves as a critical mediator linking values with attitudes, while social media advertising is a powerful external stimulus that enhances consumers' efficacy, value identification and motivational drivers. These results not only validate the effectiveness of a multi-theoretical framework but also provide actionable insights for designing marketing strategies and public policies that promote sustainable food practices in the context of sustainable food consumption.

The remainder of this paper is organized as follows: Section “Literature review and hypothesis development” provides a literature review and develops the research hypotheses. Section “Materials and methods” describes the materials and methods, including the theoretical framework and research model, questionnaire design, sample and data collection, and analysis. Section “Analysis and results” presents the analysis and results, covering the measurement model (reliability and validity), overall model fit assessment and path analyses. Section “Discussion” discusses the key findings and their theoretical and practical implications. Finally, Section “Conclusions and recommendations” concludes the study and offers recommendations for future studies.

## Literature review and hypothesis development

### Value-belief-norm theory (VBNT)

VBNT, introduced by Stern (2000), elucidates the development of environmentally supportive behaviors. It delineates the interplay between values, beliefs, and norms in shaping these behaviors. The theory underscores the significance of altruistic values, awareness of consequences (AC), ascription of responsibility (AR), and personal norms (PNs) in predicting such behaviors.

VBNT is particularly relevant to the adoption of upcycled foods because purchasing such products is not merely an economic decision but also a moral and environmental one (Chen and Chao, 2025; Moshtaghian et al., 2024). Consumers may hold positive environmental values, but without heightened awareness of environmental consequences, a sense of personal responsibility, and the activation of personal norms, such values alone are often insufficient to drive actual purchase behavior (He et al., 2021). By clarifying how values and moral norms interact to shape behavior, the VBNT directly addresses the first research question of this study: how consumers' values and beliefs translate into behavioral intentions toward upcycled food. VBNT provides a theoretical foundation that complements other perspectives, serving as the starting point for our integrated framework, which combines values, motivations, and social influences to explain consumer decision-making in this emerging sector.

#### Values

Values are categorized into egoistic, altruistic (AV), and biospheric (BV) types, each exerting distinct effects on environmental actions (De Groot and Steg, 2007). Individuals with pronounced BV and AV are more inclined to engage in environmentally friendly behaviors (Wang et al., 2021). Empirical evidence suggests that values are strong predictors of behavior (Feather, 2021). Recent research further supports the role of green consumption values in shaping sustainable food choices. For example, Kayani et al. (2023) demonstrated that families prioritizing environmentally conscious behaviors and green values are more likely to purchase organic food, underscoring the significance of values in promoting sustainability. Similarly, Fahlevi et al. (2023) found that in the Chinese green agricultural product market, consumption value, social influence, and health consciousness significantly shape consumer attitudes, which strongly predict purchase intentions. This finding reinforces the importance of values and contextual factors in influencing sustainable food choices.

While BV and AV typically promote pro-environmental actions, relying solely on these values may not suffice to alter behavior. Other motivational factors are also required (Steg et al., 2014). Sagiv and Roccas (2021) proposed that additional factors may mediate the relationship between values and behavior. Maleknia et al. (2024) identified that BVs and AC positively influence attitudes toward forest conservation, although this finding is context-specific and may not be generalizable to all environmental actions. Kim et al. (2022) demonstrated that AVs shape beliefs and attitudes regarding environmental issues; however, their behavioral impact may vary based on issue relevance and personal responsibility. Based on these insights, we propose the following hypothesis:

**Hypothesis (H1)**: *BVs have a significant positive impact on AC*.

**Hypothesis (H2)**: *AVs have a significant positive impact on AC*.

#### Beliefs

##### Awareness of consequences (AC)

AC refers to an individual's cognitive understanding of environmental issues and their potential impact. This concept incorporates comprehension and prediction of potential threats to the environment. According to Mao et al. (2020), humans are responsible for mitigating the depletion of natural resources and engaging in environmental protection behaviors such as energy-saving and carbon-reducing measures (Zeiske et al., 2021). Moreover, Zhang et al. (2020) found that the AC of Chinese farmers with respect to climate change influences their views on AR, which, in turn, affects their BIs toward climate change. This suggests that heightened AC with respect to the environment can enhance an individual's sense of responsibility for addressing environmental consequences. Based on these findings, we propose the following hypotheses:

**Hypothesis (H3):** *AC has a significant positive impact on AR*.

##### Ascription of responsibility (AR)

AR refers to an individual's recognition of their responsibility for mitigating environmental issues. Carfora et al. (2021) demonstrated that individuals perceive themselves to be responsible for addressing environmental problems and are more likely to feel compelled to adopt pro-environmental behaviors. However, it is essential to note that AR can be influenced by various contextual factors such as social norms and the perceived effectiveness of individual actions. Based on this discussion, we propose the following hypothesis:

**Hypothesis (H4):** AR has a significant positive impact on PNs.

#### Personal norms (PNs)

PNs are considered crucial drivers of environmental behavior, reflecting the sense of obligation that individuals feel to take responsible actions for the environment. According to Yan and Chai (2021), PNs are strong predictors of pro-environmental behavioral performance, as individuals who internalize these norms are more likely to engage in behaviors that align with their moral and ethical beliefs. Research has consistently shown that PNs are significantly correlated with pro-environmental BIs (Sarmento and Loureiro, 2021). Thus, we propose the following hypotheses:

**Hypothesis (H5):** PNs have a significant positive impact on BIs.

### Social cognitive theory (SCT)

Proposed by Bandura (2001), the Social Cognitive Theory (SCT) provides a framework for understanding the dynamic interactions among environmental factors, personal beliefs, and behaviors. The core constructs of SCT include self-efficacy (SE), outcome expectations, observational learning, and behavioral intention (BIs). Among these, self-efficacy is particularly crucial as it reflects individuals' confidence in their ability to perform a given behavior, thereby influencing their behavioral intentions and actual behaviors (Bandura, 2001).

In recent years, SCT has been applied to sustainability contexts. For example, Jahari et al. (2022) pointed out that SCT shares conceptual similarities with VBNT, thereby deepening the understanding of sustainable energy consumption (SEC). In the field of sustainable consumption, SCT helps explain how consumers, through exposure to social and environmental cues, such as social media marketing campaigns, can have their self-efficacy and outcome expectations shaped, which, in turn, affects their intentions to adopt sustainable products (Kumar and Pandey, 2023; Zhang, 2023). By emphasizing self-efficacy and social influence and incorporating environmental factors, SCT complements the VBNT's focus on values and norms, thereby strengthening the theoretical foundation of this study for examining consumer adoption of upcycled foods.

#### Social media advertising (SMA)

SMA plays a crucial role in influencing purchasing behavior by engaging individuals and integrating brands into their daily lives (Kumaradeepan, 2021). Jahari et al. (2022) employed campus advertisements to examine sustainable behaviors among young individuals within the SCT framework and found that SMA positively influences SE (Trejo et al., 2024). Additional research indicates that social media can elevate awareness of green consumption among young people in China, with subjective norms and perceived green values serving as mediators (Xie and Madni, 2023). Kumar and Pandey (2023) emphasized that social media can generate motivations that affect green purchasing intentions and behaviors. Zhang (2023) also identified that social media marketing can alter individuals' thoughts and emotions, thereby impacting their purchasing behavior. Based on these findings, this study proposes the following hypotheses:

**Hypothesis (H6a):** SMA has a significant positive impact on SE.

**Hypothesis (H6b):** SMA has a significant positive impact on BVs.

**Hypothesis (H6c):** SMA has a significant positive impact on AVs.

**Hypothesis (H6d):** SMA has a significant positive impact on autonomous motivation (AM).

**Hypothesis (H6e):** SMA has a significant positive impact on controlled motivation (CM).

#### Self-efficacy (SE)

SE refers to an individual's confidence in their ability to perform specific behaviors (Datsenko, 2023). Research has consistently demonstrated a positive correlation between SE and BIs, highlighting the pivotal role of SE in shaping individuals' intentions to support climate-change policies (Choi and Hart, 2021). This correlation can be attributed to the notion that individuals with higher SE tend to form strong intentions to engage in pro-environmental behaviors. Based on these findings, this study posits the following hypotheses:

**Hypothesis (H7):** SE has a significant positive impact on BIs.

### Behavioral intention (BIs)and actual behavior

The association between behavioral intentions (BIs) and actual behavior is a pivotal topic in social psychology. Robust BIs are instrumental in forecasting behavior and narrowing the gap between intentions and actions (Conner and Norman, 2022). Nevertheless, this gap often persists, is resistant to change, and is influenced by cognitive biases that can hinder action. This study proposes the following hypothesis:

**Hypothesis (H8):** BIs have a significant positive impact on actual behavior.

### Self-determination theory (SDT)

SDT provides a framework for understanding the intrinsic motivational processes that underlie human behavior (Moller et al., 2006). Positing that individuals have three innate psychological needs—relatedness, competence, and autonomy—SDT offers a nuanced explanation of the gap between intentions and actual behavior, which is discussed in detail below.

In the context of upcycled foods, SDT helps explain how different types of motivation influence consumers' purchasing intentions. When consumers are driven by autonomous motivation to purchase upcycled foods, these choices align with their environmental values or self-identity, making it more likely that they will sustain long-term sustainable consumption behaviors. In contrast, controlled motivation may temporarily promote adoption due to social pressure or marketing incentives, but it is less effective in driving long-lasting behavioral changes. Recent studies have shown that intrinsic motivation significantly promotes long-term pro-environmental behaviors (Pham et al., 2022).

SDT's value lies in its ability to complement VBNT's emphasis on values and norms and SCT's focus on social influence and self-efficacy while incorporating motivational mechanisms. This provides a more comprehensive theoretical framework for explaining consumer decision-making in the adoption of sustainable foods.

#### Autonomous motivation (AM)

AM refers to the degree to which an individual's behavior is driven by self-determination, internal willingness, and volition (Ryan and Deci, 2000a). This type of motivation encompasses intrinsic motivation, identified regulation, and integrated regulation and is characterized by a sense of autonomy and self-endorsement (Vansteenkiste et al., 2009). Empirical research has consistently found that AM is a critical predictor of goal attainment because it facilitates conscious effort and persistence (Riddell et al., 2024). Based on this theoretical and empirical foundation, this study proposes the following hypotheses:

**Hypothesis (H9):** AM has a significant positive impact on BIs.

#### Controlled motivation (CM)

CM refers to the degree to which an individual's behavior is driven by external factors, such as rewards, pressures, or expectations (Ryan and Deci, 2000a). Research has shown that AM and CM play significant roles in shaping the BIs (Yousaf et al., 2023). CM can take many forms, including knowledge seeking, escape motives, achievement, thrilling experiences, social relationships, and social media use. Using this theoretical and empirical foundation, this study proposes the following hypotheses:

**Hypothesis (H10):** CM has a significant positive impact on BIs.

## Materials and methods

### Theoretical framework and research model

**Figure 1:**
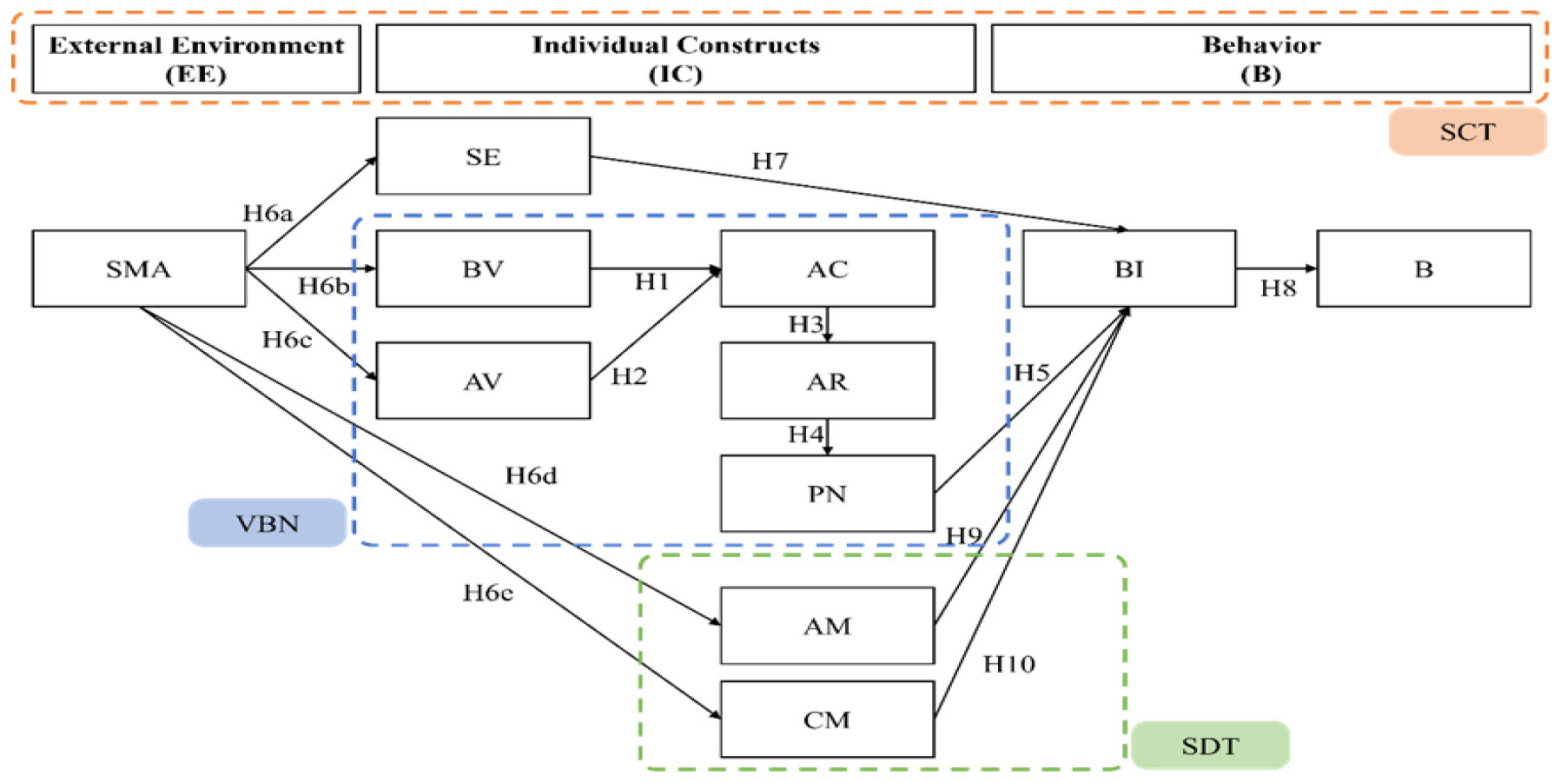
Research framework diagram. *Source* Authors own work.

This study employed a multidisciplinary approach grounded in the integration of three theoretical frameworks: VBNT, SCT, and SDT. The resulting updated food behavior evaluation model (Figure 1) provides a comprehensive framework for examining the complex interplay between internal psychological factors and external environmental influences on consumer behavior.

### Questionnaire design

The questionnaire employed in this study comprised two sections, each soliciting specific information from participants. Section 1: Measurement of Research Variables. Variables derived from VBNT theory, such as BVs, AVs, AC, AR, and PNs, were evaluated using a 17-item scale. This scale was based on studies by Sun et al. (2024), Sánchez-García et al. (2021), Kim et al. (2022), Al Mamun et al. (2024), Chen (2024), and Carfora et al. (2021). Variables associated with SCT, including SMA, SE, BIs, and actual behavior, were measured using a 14-item scale based on studies by Wibowo et al. (2021), Shahangian et al. (2021), Lin et al. (2020), and Ma and Shen (2024). Variables from SDT, such as AM and CM, were assessed using a seven-item scale based on Ma and Shen's (2024) research. All items used a 7-point Likert scale, with 1 indicating “strongly disagree” and 7 indicated “strongly agree.” Higher scores reflect a more positive perception of attributes.

Section 2: Demographic Data: This section gathered demographic data, including sex, age, education level, monthly income, occupation, and dietary habits.

### Sample and data collection

The research team referred to the relevant literature and expert suggestions to develop items consistent with the research framework, covering several theoretical dimensions. To ensure the reliability and validity of the questionnaire, a pilot test was conducted by distributing the survey link via various social media platforms and inviting 50 participants from diverse backgrounds to complete it. Reliability analysis showed that most Cronbach's α values exceeded 0.7, indicating good internal consistency. Based on the factor analysis results, items with factor loadings below 0.5 were removed; for example, the altruistic value construct was reduced from four items to three, thereby improving the scale's validity. Importantly, no particular construct proved especially difficult to measure, and the adapted scales generally performed consistently with findings from prior studies. The minor refinement of the altruistic value construct illustrates that while the measurement instruments were largely robust in the Taiwanese context, small adjustments were necessary to optimize psychometric performance.

Formal data collection was conducted from January 1 to March 1, 2025. The research team distributed the questionnaire link through multiple social media platforms, such as Facebook, Instagram, and Line groups, and encouraged respondents to share the link via word-of-mouth to increase the response rate. Before completing the questionnaire, the participants were required to read an electronic consent form that provided detailed information about the study's purpose, procedures, ethical considerations, data confidentiality measures, and participants' rights. The form emphasized that participation was entirely voluntary to ensure that the respondents fully understood and willingly agreed to participate in the study.

The study employed anonymous responses and did not collect any personally identifiable information to protect the participants' privacy. After data collection, the research team applied predefined screening criteria and excluded questionnaires that were incomplete or exhibited obvious patterns of inconsistent responses. In total, 460 questionnaires were obtained. After excluding incomplete or invalid responses, 402 valid responses remained, yielding an 87.39% valid response rate. Table 1 presents the demographic characteristics of the participants. The 402 valid responses exhibited a nearly equal gender distribution: 49.5% were male and 50.5% were female. The largest age group was 21–30 years (28.4%), followed by 51–60 years (24.6%). In terms of education, 42.0% held a senior high/vocational degree and 37.1% held a college or university degree. The majority (43.8%) reported earnings between NT$20,001 and NT$40,000. The sample encompassed various occupations, with the service industry representing the largest group (29.9%).

**Table 1:** Demographic analysis examines the characteristics of a population.

***N* = 402**	**Item**	**Population**	**Percentage (%)**
Sex	Male	199	49.5
	Female	203	50.5
Age	21–30 years	114	28.4
	31–40 years old	91	22.6
	41–50 years old	90	22.4
	51–60 years	99	24.6
	60 years and above	8	2.0
Education level	Junior high or below	35	8.7
	Senior high/vocational	169	42.0
	College/university	149	37.1
	Master's or above	49	12.2
Personal monthly income	Less than NT$20,000	41	10.2
	NT$20,001–40,000	176	43.8
	NT$40,001–60,000	73	18.2
	NT$60,001–80,000	56	13.9
	NT$80,001–100,000	24	6.0
	Above NT$100,001	32	8.0
Occupation	Student	24	6.0
	Army, civil service, and education	49	12.2
	Service industry	120	29.9
	Freelance	33	8.2
	Traditional manufacturing	72	17.9
	Specialized occupation (e.g., doctor and lawyer)	16	4.0
	Other	88	21.9

NTD, New Taiwan dollar (1 NTD = 0.030 USD).

Source: Authors own work.

### Data analysis methods

Quantitative analyses were performed using SPSS and AMOS software. Initially, descriptive statistics were used to characterize the sample. Subsequently, reliability and confirmatory factor analyses were performed to evaluate the internal consistency and validity of the measurement model. Structural equation modeling (SEM) was then applied to test the hypotheses and investigate the causal relationships among variables.

## Analysis and results

### Measurement model: reliability and validity

This study employed a two-step methodology to evaluate the reliability and validity of the model. Initially, a CFA was conducted to examine the relationship between the observed variables and latent constructs, ensuring that the observed variables accurately represented the latent constructs.

The CFA results indicated that the standardized factor loadings for each concept ranged from 0.650 to 0.965, demonstrating strong explanatory power and indicating that the items for each concept represented the latent variables effectively.

Composite Reliability (CR) was calculated to assess the consistency of each construct, with CR values ranging from 0.832 to 0.965, all exceeding the 0.60 threshold recommended by Fornell and Larcker (1981), indicating excellent internal consistency. Additionally, the Average Variance Extracted (AVE) was calculated to assess convergent validity, with AVE values ranging from 0.627 to 0.903, all surpassing the 0.50 standard, indicating good convergent validity.

Cronbach's α was calculated for each construct to assess reliability, with results ranging from 0.661 to 0.946. Most constructs exhibited values above 0.70, indicating a high reliability. One construct exhibited a value between 0.30 and 0.70, indicating moderate reliability, yet remained acceptable for further analysis.

Detailed data for each construct's factor loadings, CR, AVE, and Cronbach's α are presented in Table 2. Overall, the results demonstrate that the measurement model employed in this study exhibited high internal consistency and convergent validity, thereby ensuring the reliability and validity of the tool.

**Table 2:** Outcomes regarding factor loading, reliability, and validity.

**Variables**	**Items**	**Standardized factor loading**	**CR**	**AVE**	**Cronbach's α**
Biospheric Value (BV)	1. I believe that it is my responsibility to protect the natural environment.	0.927^***^	0.944	0.808	0.916
	2. I consider myself an integral part of nature.	0.872^***^			
	3. I believe that we should respect Earth and its ecosystems.	0.937^***^			
	4. I think I should share my efforts in improving the world.	0.858^***^			
Altruistic Value (AV)	5. I believe that all the people were born equally.	0.774^***^	0.852	0.658	0.712
	6. I think the world should achieve peace.	0.865^***^			
	7. I believe that society should be based on justice.	0.820^***^			
Awareness of Consequences (AC)	8. I consider global warming a significant social issue today.	0.859^***^	0.832	0.627	0.661
	9. I think that choosing sustainable beer helps improve environmental problems.	0.849^***^			
	10. I believe that environmental protection can enhance quality of life.	0.650^***^			
Ascription of Responsibility (AR)	11. I think everyone should take responsibility for the environmental problems caused by food waste.	0.817^***^	0.910	0.772	0.851
	12. I believe that everyone should share responsibility for the environmental impact of their food choices.	0.901^***^			
	13. I think everyone is responsible for the environmental problems caused by food choice.	0.914^***^			
Personal Norm (PN)	14. I believe that I should choose to buy sustainable beer to protect the environment.	0.896^***^	0.941	0.842	0.905
	15. I think I have a moral obligation to buy sustainable beer to maintain Earth's environment.	0.931^***^			
	16. I believe that I should prioritize buying sustainable beer over traditional beer to protect the environment.	0.925^***^			
Social Media Advertising (SMA)	17. I would buy sustainable beer from online stores on social media because the content is attractive.	0.909^***^	0.933	0.737	0.910
	18. I would buy sustainable beer from online stores on social media because it provides the latest product information.	0.902^***^			
	19. I would buy sustainable beer from online stores on social media because it offers customized information search functions.	0.864^***^			
	20. I am willing to share information about brands and products/services with my friends on social media.	0.804^***^			
	21. I am willing to upload or forward content from online stores to my social media platforms.	0.808^***^			
Self-Efficacy (SE)	22. If I want to support sustainable development by buying sustainable beer, it will be absolutely feasible for me.	0.856^***^	0.909	0.770	0.792
	23. For me, supporting sustainable development by buying sustainable beer was relatively easy.	0.903^***^			
	24. Whether I choose to buy sustainable beer to support my sustainable development depends entirely on me.	0.872^***^			
Behavioral Intention (BI)	25. If I know that upcycled food is beneficial to the environment, I will choose to buy it.	0.765^***^	0.903	0.757	0.848
	26. When the quality of upcycled food is equivalent to that of traditional food, I consider buying upcycled food.	0.895^***^			
	27. If I have the opportunity to choose upcycled food, I plan to incorporate it in my diet.	0.940^***^			
Behavior (B)	28. I often choose to buy upcycled food.	0.957^***^	0.965	0.903	0.946
	29. I often buy upcycled food from nearby stores.	0.965^***^			
	30. I often choose upcycled food and pay attention to whether its packaging meets the sustainable development standards.	0.928^***^			
Autonomous Motivation (AM)	31. If I take green actions for the environment, the sense of accomplishment from improving environmental quality will bring me joy.	0.863^***^	0.919	0.739	0.875
	32. I believe that taking care of myself and protecting the environment are inseparable, so I actively take green actions that benefit the environment.	0.926^***^			
	33. I believe that taking green action in the environment is a wise decision.	0.852^***^			
	34. If I do not take any action in the environment, I feel guilty.	0.792^***^			
Controlled Motivation (CM)	35. If I do not take any action in the environment, people around me may feel dissatisfied.	0.878^***^	0.937	0.833	0.896
	36. If I take green actions in the environment, I can gain recognition and affirmation from others.	0.930^***^			
	37. If I take green actions in the environment, I can avoid criticism or blame from others.	0.929^***^			

^***^ p <0.001.

Source: Authors own work.

The results in Table 3 indicate that the square root of the AVE for each construct was higher than its correlation with other constructs. This finding demonstrates good discriminant validity, indicating that the tool can effectively differentiate between distinct latent variables. This supports the reliability and validity of the model, and enhances the internal and external validity of the findings.

**Table 3:** Correlation coefficients alongside the square root of AVE.

	**Mean**	**Standard deviation**	**1**	**2**	**3**	**4**	**5**	**6**	**7**	**8**	**9**	**10**	**11**
1.BV	6.0498	1.1658	**0.899**										
2.AV	5.8864	1.1449	0.264^**^	**0.811**									
3.AC	6.1667	0.8859	0.410^**^	0.550^**^	**0.792**								
4.AR	6.4163	0.7443	0.472^**^	0.369^**^	0.629^**^	**0.878**							
5.PN	5.2280	1.2687	0.214^**^	0.432^**^	0.641^**^	0.522^**^	**0.917**						
6.SMA	5.2692	1.1960	0.220^**^	0.441^**^	0.624^**^	0.429^**^	0.817^**^	**0.859**					
7.SE	5.6318	0.9965	0.269^**^	0.286^**^	0.438^**^	0.511^**^	0.582^**^	0.557^**^	**0.877**				
8.BI	5.6849	0.9869	0.505^**^	0.319^**^	0.410^**^	0.534^**^	0.574^**^	0.494^**^	0.658^**^	**0.870**			
9.B	3.9030	1.7279	0.198^**^	0.285^**^	0.506^**^	0.261^**^	0.652^**^	0.663^**^	0.374^**^	0.391^**^	**0.950**		
10.AM	5.4198	1.0812	0.544^**^	0.444^**^	0.506^**^	0.524^**^	0.617^**^	0.580^**^	0.538^**^	0.787^**^	0.455^**^	**0.860**	
11.CM	4.7504	1.2812	0.398^**^	0.368^**^	0.453^**^	0.435^**^	0.679^**^	0.755^**^	0.490^**^	0.675^**^	0.605^**^	0.769^**^	**0.913**

The bold values on the diagonal represent the square root of AVE for each variable.

^**^ p <0.01.

Source: Authors own work.

### Overall model fit assessment

To assess the adequacy of the proposed model, an overall model fit assessment was conducted using maximum likelihood (ML) estimation. The study yielded the following results: χ^2^/d*f* = 4.227 (acceptable), Comparative Fit Index (CFI) = 0.637 (acceptable), Standardized Root Mean Square Residual (SRMR) = 0.077 (acceptable), and Root Mean Square Error of Approximation (RMSEA) = 0.063 (indicating good fit). Additionally, the Tucker–Lewis Index (TLI) was 0.962, exceeding the threshold of 0.90 (Kline, 2023), indicating a parsimonious and robust model.

### Overall model path analysis

**Figure 2:**
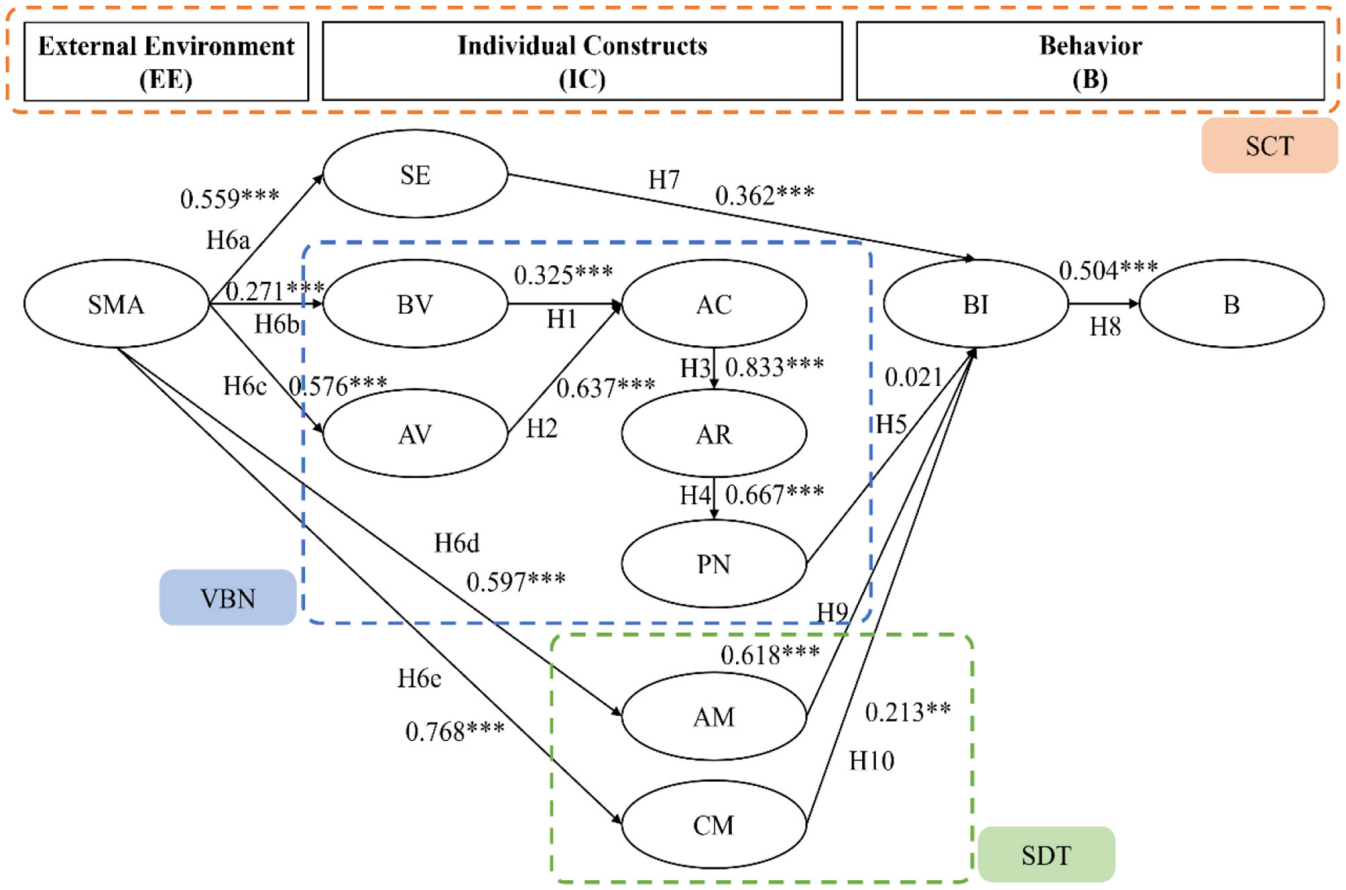
Structural equation modeling diagram. **p < 0.01; ***p < 0.001. *Source* Authors own work.

This study employed structural equation modeling to examine the interrelationships among the constructs. The structural model analysis diagram is shown in Figure 2.

The results of the path analysis and hypothesis testing are summarized in Table 4. The findings indicate that BVs have a statistically significant and positive impact on AC (H1: β = 0.325, *p* < 0.001), suggesting that individuals who prioritize environmental wellbeing are more likely to be aware of the consequences of their actions. Similarly, AVs were found to have a significant and positive impact on AC (H2: β = 0.637, *p* < 0.001), indicating that individuals who prioritize others' wellbeing are more likely to be aware of the consequences of their actions. These results are consistent with prior studies emphasizing the role of biospheric and altruistic values in shaping environmental awareness (Steg et al., 2014; Dhir et al., 2025).

**Table 4:** Findings from the path analysis and validations of hypotheses.

**Hypothesized paths**	**Unstandardized coefficient**	**S.E**.	**C.R**.	***p***	**Standardized coefficients(β)**	**Verification results**
H1:BV → AC	0.480	0.077	6.203	^***^	0.325	Positive and significant
H2:AV → AC	0.800	0.098	8.181	^***^	0.637	Positive and significant
H3:AC → AR	0.979	0.116	8.423	^***^	0.833	Positive and significant
H4:AR → PN	0.495	0.049	10.106	^***^	0.667	Positive and significant
H5:PN → BI	0.051	0.128	0.401	0.689	0.021	Insignificant
H6a:SMA → SE	0.674	0.078	8.657	^***^	0.559	Positive and significant
H6b:SMA → BV	0.282	0.056	5.028	^***^	0.271	Positive and significant
H6c:SMA → AV	0.705	0.092	7.694	^***^	0.576	Positive and significant
H6d:SMA → AM	0.744	0.071	10.402	^***^	0.597	Positive and significant
H6e:SMA → CM	1.199	0.096	12.476	^***^	0.768	Positive and significant
H7:SE → BI	0.964	0.252	3.819	^***^	0.362	Positive and significant
H8:BI → B	0.181	0.034	5.319	^***^	0.504	Positive and significant
H9:AM → BI	1.595	0.330	4.836	^***^	0.618	Positive and significant
H10:CM → BI	0.439	0.138	3.186	^**^	0.213	Positive and significant

^***^p <0.001; ^**^p <0.01.

Source: Authors own work.

The results also show that AC has a significant and positive impact on AR (H3: β = 0.833, *p* < 0.001), suggesting that individuals who are aware of the consequences of their actions are more likely to attribute responsibility to themselves. This aligns with previous work showing that awareness of environmental consequences increases individuals' sense of responsibility (Caniëls et al., 2021). Furthermore, AR was found to have a significant and positive impact on PNs (H4: β = 0.667, *p* < 0.001), indicating that individuals who attribute responsibility to themselves are more likely to develop PNs to guide their behavior. This supports the central assumptions of the Value-Belief-Norm theory (Stern, 2000), confirming that responsibility attribution fosters moral norms.

However, these findings did not support the hypothesis that PNs have a significant impact on BIs (H5: β = 0.021, *p* = 0.689). This suggests that PNs may not be a direct predictor of BIs and other factors may play a more significant role in shaping an individual's intention to engage in environmentally friendly behavior. This differs from earlier findings where personal norms were strong predictors of pro-environmental intentions (Harland et al., 1999; Sarmento and Loureiro, 2021).

The results also underline the significant and positive impact of SMA on SE (H6a: β = 0.559, *p* < 0.001), BVs (H6b: β = 0.271, *p* < 0.001), AVs (H6c: β = 0.576, *p* < 0.001), AM (H6d: β = 0.597, *p* < 0.001), and CM (H6e: β = 0.768, *p* < 0.001). These findings suggest that SMA can be an effective tool for promoting environmentally friendly behavior by influencing individuals' values, motivation, and SE. These results echo prior studies that highlight the role of social media in enhancing values, efficacy, and motivation for sustainable behaviors (Xie and Madni, 2023; Kumar and Pandey, 2023).

The results also show that SE has a significant and positive impact on BIs (H7: β = 0.362, *p* < 0.001), indicating that individuals who believe in their ability to make a difference are more likely to engage in environmentally friendly behavior. This is consistent with the findings of Lin et al. (2020), who reported that higher self-efficacy strengthens pro-environmental behavioral intentions. In addition, BIs were found to have a significant and positive impact on actual behavior (H8: β = 0.504, *p* < 0.001), suggesting that individuals who intend to engage in environmentally friendly behaviors are more likely to do so. This finding aligns with the Theory of Planned Behavior (Ajzen, 1991) and other empirical studies confirming the intention–behavior link (Jiang et al., 2023).

Finally, the findings indicate that AM has a significant and positive impact on BIs (H9: β = 0.618, *p* < 0.001), suggesting that individuals motivated by autonomy are more likely to engage in environmentally friendly behaviors. CM also had a significant and positive impact on BIs (H10: β = 0.213, *p* < 0.001), although its effect size was smaller than that of AM. These results are consistent with Self-Determination Theory (Ryan and Deci, 2000b) and resonate with Yousaf et al. (2023), who found that autonomous motivation exerts a stronger influence on sustainable consumption than controlled motivation.

Most hypotheses were supported in the analysis, highlighting the significant impact of values, motivation, SE, and SMA on consumers' BIs toward upcycled food. However, an interesting finding emerged with respect to H5, which proposed that PNs would have a positive effect on BIs. Contrary to expectations, the results show that PNs do not have a significant influence on consumers' BIs toward upcycled food (β = 0.08, *p* > 0.05). This nonsignificant result is further discussed below to explore the possible reasons for this unexpected result and their implications for the research.

## Discussion

Our findings highlight the significant impact of values, SMA, SE, and motivation on consumers' BIs, with a focus on the unexpected finding that PNs do not significantly influence their intentions. This mixed outcome underscores the complexity of theoretical integration and the context-dependent nature of consumer behavior. This result also suggests that when the market is still in its emerging stage, certain theoretical constructs (such as PNs) may fail to demonstrate the same explanatory power as they do in mature markets, reminding us that the applicability of existing theories across cultures or different stages of market development should be questioned.

Notably, our findings support the central tenets of the VBNT theory. In particular, we found that BVs and AVs positively influenced AC (H1 and H2), which is consistent with the expectations of Dhir et al. (2025) and Caniëls et al. (2021). Furthermore, AC mediated the relationship between values and AR (H3), whereas AR significantly affected PNs (H4). These results underscore the importance of environmental responsibility and moral awareness in consumer decision making. However, whether the strength of these relationships remains equally robust across different cultural contexts or sustainability issues warrants further examination.

According to Sarmento and Loureiro (2021), PNs have a positive impact on pro-environmental BIs, suggesting that they are linked to the formation of moral obligations that typically stem from formal and informal education. However, this study found that PNs did not significantly influence Taiwanese consumers' BIs toward upcycled food. This result can be explained from two perspectives: the market development stage and consumer awareness level. Viewed more broadly, this may reflect that Taiwanese consumers have not yet internalized upcycled food as a consumption choice grounded in moral obligation but instead regard it as a novel or fashionable product.

First, the upcycled food market in Taiwan remains in a developmental stage, meaning that the market has not yet sufficiently matured to profoundly influence consumers' sense of moral responsibility and behavioral norms. In this study's sample, most consumers lacked knowledge of upcycled food, making it difficult for them to establish a strong connection between upcycled food and moral obligations in the decision-making process. As Sarmento and Loureiro (2021) pointed out, consumers' moral obligations often stem from education, whether formal education in schools or informal education through social media or at home. However, consumers who lack awareness of upcycled food may not have been sufficiently exposed to relevant education or advocacy, making it difficult to link upcycled food consumption with environmental responsibility and moral duties. Consequently, the role of PNs in this process is limited, which leads to a smaller impact on consumer BIs.

However, this phenomenon can also be interpreted through the lens of the “attitude–behavior gap” widely discussed in sustainable consumption research. This literature highlights that even when individuals hold strong environmental values or positive attitudes, their actions may not fully align because of contextual and structural barriers such as high prices, lack of information, or immature market conditions (Vieira et al., 2023; Bocti et al., 2021). In the case of upcycled foods in Taiwan, consumers may conceptually support sustainability but fail to translate this support into intentions or behaviors, as the necessary conditions to activate personal norms are not yet present. This connection helps to contextualize why PNs did not emerge as a significant predictor in this study, despite their established role in previous studies.

Second, while PNs are important for pro-environmental behavior, other factors such as SE and AM may play a more prominent role in shaping consumers' BIs in the current context. This suggests that consumers' understanding and acceptance of upcycled food are influenced by their perceptions of the product's effectiveness and SE. If consumers believe that they can easily obtain and understand these products and feel capable of consuming them, they are more likely to make decisions in favor of such foods. This indicates that, compared to internalized moral obligations, current consumers rely more on self-efficacy and external motivations to drive their decisions, indicating that sustainable consumption in Taiwan remains at a relatively superficial stage. This finding underscores the need for enhanced education and advocacy efforts in the current upcycled food market, particularly to increase consumer awareness of the product and its environmental value. As the market matures and educational resources expand, consumers may gradually link upcycled food with environmental responsibility and moral obligations, thereby increasing the influence of PNs on BIs.

This study found that the specific context of the upcycled food market in Taiwan and consumers' limited product knowledge prevented PNs from having a significant impact. This result challenges Sarmento and Loureiro's conclusions and suggests that in an immature market, other psychological factors may have a more prominent influence on consumers' BIs.

SMA has emerged as a critical factor in shaping multiple constructs including SE, values, and motivations (H6a–H6e). This result aligns with those of Xie and Madni (2023), Kumar and Pandey (2023), and Zhang (2023), highlighting the important role of social media in promoting sustainable consumption behavior. The omnipresence of social media in contemporary consumer culture underscores the need to acknowledge its influence on consumer behavior.

Our study also supports the SCT and SDT frameworks, demonstrating that SE (H7), AM (H9), and CM (H10) significantly influence BIs. A noteworthy finding is the differential impact of autonomy (β = 0.618) and CM (β = 0.213) on BIs. This suggests that when consumers perceive that purchasing upcycled food aligns with their internal values, their BIs is more pronounced. This result echoes that of Yousaf et al. (2023) and underscores the importance of internal motivation in driving sustainable consumption behaviors. This disparity reminds us that if strategies for promoting sustainable consumption rely too heavily on external norms or constraints, their effectiveness may be limited; only when consumers genuinely connect their behaviors with their internal values can lasting behavioral change be achieved.

Finally, our study validated the effectiveness of the theoretical model, demonstrating a significant relationship between BIs and actual behavior (H8). This outcome is consistent with that of Jiang et al. (2023), Amanda and Marsasi (2024), and Ajzen (1991), who underscored the value of BIs as a predictor of behavior. Our findings emphasize the need to recognize the interplay between values, SMA, SE, and motivation in shaping consumer behavior toward upcycled food.

Notably, the finding that PNs have no significant impact on BIs (H5) suggests that this relationship warrants further investigation. A deeper exploration of this phenomenon could provide valuable insights into the complex dynamics influencing consumer behavior and inform the development of more effective strategies for promoting sustainable consumption in the future.

This study contributes to a deeper understanding of the factors that drive consumer behavior in an emerging upcycled food market, and provides actionable insights for marketers, policymakers, and researchers. To promote sustainable consumption, businesses should prioritize environmental and social values to enhance consumers' AC in their actions and sense of responsibility. SMA can serve as an effective strategic tool to increase brand awareness and influence consumer values and BIs.

Policymakers should focus on strengthening consumers' AM through targeted educational and promotional programs that highlight the environmental benefits of upcycled food. The implementation of such strategies can foster the development of an upcycled food market and promote sustainable consumption behaviors.

## Conclusions and recommendations

This study utilized PLS-SEM to verify the factors that influence consumers' BIs toward upcycled food and revealed that values, SE, and motivation are crucial variables influencing both BIs and actual behavior. BV and AV increase consumers' awareness of the environmental and social benefits of upcycled food, thereby enhancing their sense of responsibility. In addition, the role of social media advertising cannot be ignored. It not only boosts consumers' self-efficacy, but also promotes their value identification with upcycled foods and strengthens their autonomy and control motivations. These factors collectively drive consumer purchasing behavior.

Theoretically, this study validates the effectiveness of integrating VBNT, SCT, and SDT to provide a more holistic framework for explaining sustainable food consumption. Practically, the findings suggest that businesses should emphasize environmental and social values in their communication strategies and leverage social media advertising to enhance consumer motivation and effectiveness. Policymakers should design targeted education and advocacy programs to strengthen consumer awareness of the environmental benefits of upcycled foods, thereby fostering sustainable food practices.

This study had several limitations. First, the sample was restricted to Taiwanese consumers, which may affect the generalizability of the findings to other cultural contexts and nations. Importantly, the cultural specificity of Taiwan must be acknowledged in this study. For instance, the collectivist orientation of Taiwanese society may shape the way altruistic values are expressed, while the dominance of Line as the primary social media platform contrasts with Western contexts, where Facebook plays a more central role. These cultural and digital ecosystem differences may influence the way values, norms, and social influences affect behavioral intentions in different countries. Rather than being a limitation, this highlights a fruitful avenue for future cross-cultural research to examine how different cultural settings shape sustainable food adoption.

Second, most participants had limited knowledge of upcycled foods, which may have influenced the non-significant role of personal norms in the study's findings. Third, reliance on self-reported survey data raises the possibility of social desirability bias in the responses. Finally, this study measured “actual behavior” through self-reported survey items, which may be subject to social desirability bias or recall error. Although this approach is common in social science research, it remains an important limitation that should be emphasized in future studies.

Future research directions include exploring the heterogeneity of different consumer groups using methodologies such as cluster analysis, which can be adopted to examine the influence of PNs on specific groups. Experimental designs can also be used to verify the impact of SMA on consumer BIs through diverse mechanisms, thus enhancing the practical value of the research results. In addition, future studies should seek to improve measurement validity by employing behavioral tracking or experimental observations to more accurately capture consumers' actual behaviors.

## Data Availability

The original contributions presented in the study are included in the article/supplementary material, further inquiries can be directed to the corresponding author.
